# Identification of potential crucial genes and pathways associated with vein graft restenosis based on gene expression analysis in experimental rabbits

**DOI:** 10.7717/peerj.4704

**Published:** 2018-05-16

**Authors:** Qiang Liu, Xiujie Yin, Mingzhu Li, Li Wan, Liqiao Liu, Xiang Zhong, Zhuoqi Liu, Qun Wang

**Affiliations:** 1 Jiangxi Medical College, Nanchang University, Nanchang, Jiangxi Province, China; 2 Department of Cardiovascular Surgery, Cardiovascular Research Institute Laboratory, First Affiliated Hospital of Nanchang University, Nanchang, Jiangxi Province, China; 3 Department of Biochemistry and Molecular Biology, School of Basic Medical Sciences, Nanchang University, Nanchang, Jiangxi Province, China

**Keywords:** Occlusive artery disease, Vein graft restenosis, Microarray data, Differentially expressed gene, Bioinformatics analysis

## Abstract

Occlusive artery disease (CAD) is the leading cause of death worldwide. Bypass graft surgery remains the most prevalently performed treatment for occlusive arterial disease, and veins are the most frequently used conduits for surgical revascularization. However, the clinical efficacy of bypass graft surgery is highly affected by the long-term potency rates of vein grafts, and no optimal treatments are available for the prevention of vein graft restenosis (VGR) at present. Hence, there is an urgent need to improve our understanding of the molecular mechanisms involved in mediating VGR. The past decade has seen the rapid development of genomic technologies, such as genome sequencing and microarray technologies, which will provide novel insights into potential molecular mechanisms involved in the VGR program. Ironically, high throughput data associated with VGR are extremely scarce. The main goal of the current study was to explore potential crucial genes and pathways associated with VGR and to provide valid biological information for further investigation of VGR. A comprehensive bioinformatics analysis was performed using high throughput gene expression data. Differentially expressed genes (DEGs) were identified using the R and Bioconductor packages. After functional enrichment analysis of the DEGs, protein–protein interaction (PPI) network and sub-PPI network analyses were performed. Finally, nine potential hub genes and fourteen pathways were identified. These hub genes may interact with each other and regulate the VGR program by modulating the cell cycle pathway. Future studies focusing on revealing the specific cellular and molecular mechanisms of these key genes and pathways involved in regulating the VGR program may provide novel therapeutic targets for VGR inhibition.

## Introduction

Occlusive artery disease is a major cause of morbidity and mortality worldwide (Bansilal, Castellano & Fuster, 2015). Despite development of novel treatments in past decades, coronary artery bypass graft (CABG) surgery remains the standard of care for patients with left main coronary artery disease (CAD) and three-vessel CAD (Serruys et al., 2009). In contrast, most patients with late-stage peripheral artery occlusive disease are treated with peripheral artery bypass graft surgery (Weintraub et al., 2012). Due to their advantages in availability and length, veins are the most commonly used conduits in coronary and peripheral artery vascular surgeries (Goldman et al., 2004). However, one major issue that has dominated the field for many years is that autologous veins are especially prone to failure. Although data have shown that the patency rates of vein grafts diminish from 98% immediately after surgery to <88% within the first month post-surgery and to 60% at 10 years after surgery. To date, no unequivocally effective treatments are available for vein graft failure (Elmore et al., 2016). Thus, clarifying the cellular and molecular mechanisms involved in VGR and identifying potential novel therapeutic targets for the prevention of restenosis are significant goals.

At present, vein grafts are thought to undergo an adaptation process in new arterial environments during which intimal thickening and structural vessel wall remodeling appear (Buccheri et al., 2016; De Vries et al., 2016). However, the cellular and molecular mechanisms underlying intimal hyperplasia and the vascular remodeling process are largely unknown. Several studies have identified individual genes involved in restenosis; for instance, early growth response protein 1 (Egr-1) was suggested to promote endothelial cell(EC) proliferation and induce vein graft restenosis by up-regulating ICAM-1 expression (Zhang et al., 2013). Tumor necrosis factor alpha-stimulated gene 6 (TSG-6) was indicated to suppress restenosis of vein grafts in rats by inhibiting the inflammatory response (Zhang et al., 2017). Despite the rapid development of genome sequencing and microarray technologies, very few studies have characterized genes and pathways associated with VGR at the transcriptome level, which may be partly due to the challenge of obtaining appropriate samples and establishing animal models.

Kalish et al.(2004) identified the transcriptome responses of canine vein bypass grafts using transcriptional profiling and defined the individual contributions of ECs and VSMCs in a cell-specific vein graft transcriptome analysis (Bhasin et al., 2012). However, a main weakness of the former study was the unavailability of canine-specific gene arrays in 2004, as mentioned by the authors (Bhasin et al., 2012). The most important limitation of the latter study lies in the fact that only the role of ECs and VSMCs were taken into consideration. However, other cells and molecular components, such as inflammatory cells and immune cells, also play pivotal roles in mediating VGR (De Vries et al., 2016).

The purpose of the current study was to determine potential crucial genes and pathways involved in VGR. A bioinformatics analysis was performed with gene expression data from our previous study (Wang et al., 2016), which avoided the limitations of the aforementioned microarray data. After screening differentially expressed genes (DEGs) and performing a functional enrichment analysis, protein-protein interaction (PPI) networks of the DEGs were constructed and visualized. The present study makes notable contributions to our understanding of the molecular mechanisms involved in VGR and provides valuable biological information for further exploration of potential candidate biomarkers and therapeutic targets for the prevention of VGR.

## Materials & Methods

### Microarray data

The gene expression data used in this study were based on the Agilent GPL7083 platform (Agilent Rabbit 4x44K Gene Expression Microarrays). The whole rabbit genome oligo microarray provides a broad view by representing all known genes and transcripts in the rabbit genome. Sequences were compiled from a broad source survey and then verified and optimized by alignment to the assembled rabbit genome. As previously described, significant vascular wall thickening and a high level of cell proliferation were observed, whereas cell apoptosis was at the lowest level, in seven days after surgery group (Wang et al., 2016). Hence, seven days after surgery was considered a key time point for vein graft restenosis. Based on these observations, we compared the day 7 group with the control group as the best choice to identify DEGs involved in VGR. Raw microarray data (GSE110398) produced in our previous study were remodeled and further analyzed. A total of 10 samples were extracted from our previous dataset (Wang et al., 2016), including six vein graft samples (C1, C2, C3, C4, C5, and C6) removed seven days after surgery and four vein graft samples (O1, O2, O3, and O4) obtained from the sham surgery group (data can also be found in the Supplemental File). Herein, samples obtained seven days after surgery were defined as the surgery group, and samples obtained from the sham surgery group were defined as the control group.

### Identification of DEGs

The raw signal intensities were normalized with the quantile method by GeneSpring GX v11.5, and low intensity genes were filtered. The probe quality control was determined using principal component analysis (PCA) in GeneSpring. The statistical software R (version 3.4.1; R Core Team, 2017) and the linear models for microarray data package (limma) (Ritchie et al., 2015) in Bioconductor (http://www.bioconductor.org/) were applied to identify DEGs by comparing expression values between samples in the surgery and control groups. An empirical Bayes method was used to select significant DEGs based on the “limma” package in Bioconductor. In this study, we were interested in determining which genes were expressed at different levels between the control and surgery groups. In our analysis, linear models are fitted to the data with the assumption that the underlying data are normally distributed. Therefore, a design matrix was set up with the vein graft tissue information.

### Enrichment analyses of DEGs

The Database for Annotation, Visualization and Integrated Discovery (DAVID) v6.8 is an integrated functional annotation tool for investigators to examine the biological meanings underlying a large list of genes (Huang, Sherman & Lempicki, 2009). GO (gene ontology) (Gene Ontology Consortium, 2006) and KEGG (Kyoto Encyclopedia of Genes and Genomes) (Kanehisa & Goto, 2000) pathway enrichment analyses of DEGs were performed using the authoritative online tool DAVID (https://david.ncifcrf.gov/). An adjusted *p*-value <0.05 and count ≥2 were considered to have achieved significant enrichment.

### PPI network construction

The Search Tool for the Retrieval of Interacting Genes (STRING) database is an online tool designed to provide a critical assessment and integration of protein-protein interactions, including both direct (physical) and indirect (functional) associations (Szklarczyk et al., 2014). To evaluate the potential relationships among DEGs, we mapped the DEGs into STRING; only experimentally validated interactions with a combined score >0.4 were considered significant. Moreover, the Molecular Complex Detection (MCODE) app was used to identify modules of the PPI network in Cytoscape (Shannon et al., 2003) with a degree cutoff = 2, node score cutoff = 0.2, k-core = 2, and max. depth = 100. MCODE scores >3 and a number of edges >40 were set as the cut-off criteria. Pathway analysis of genes in each module was performed using the online tool DAVID, and *p*-values <0.05 were considered significant.

## Results

### Identification of DEGs

**Figure 1:**
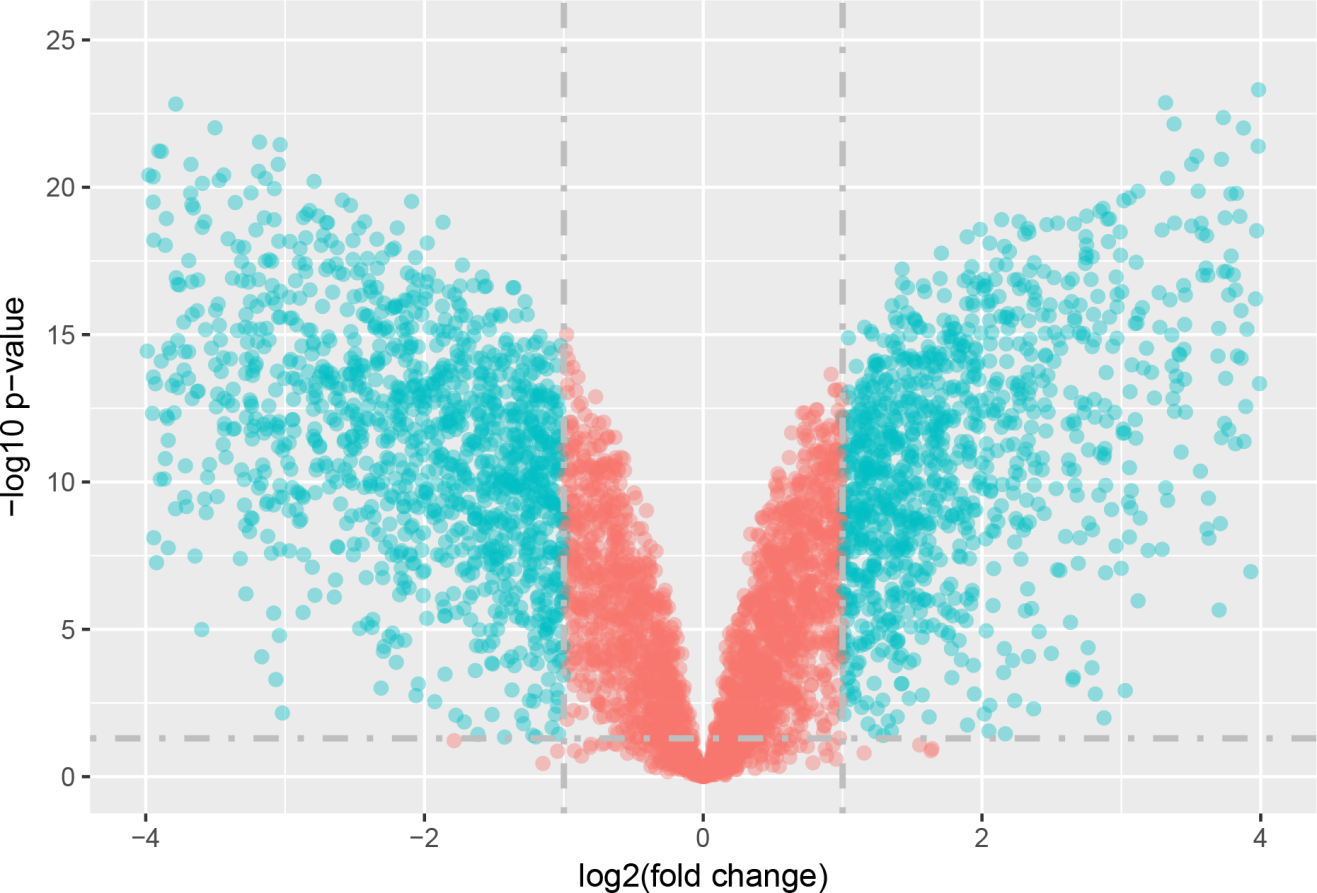
Volcano plot of DEGs.

**Figure 2:**
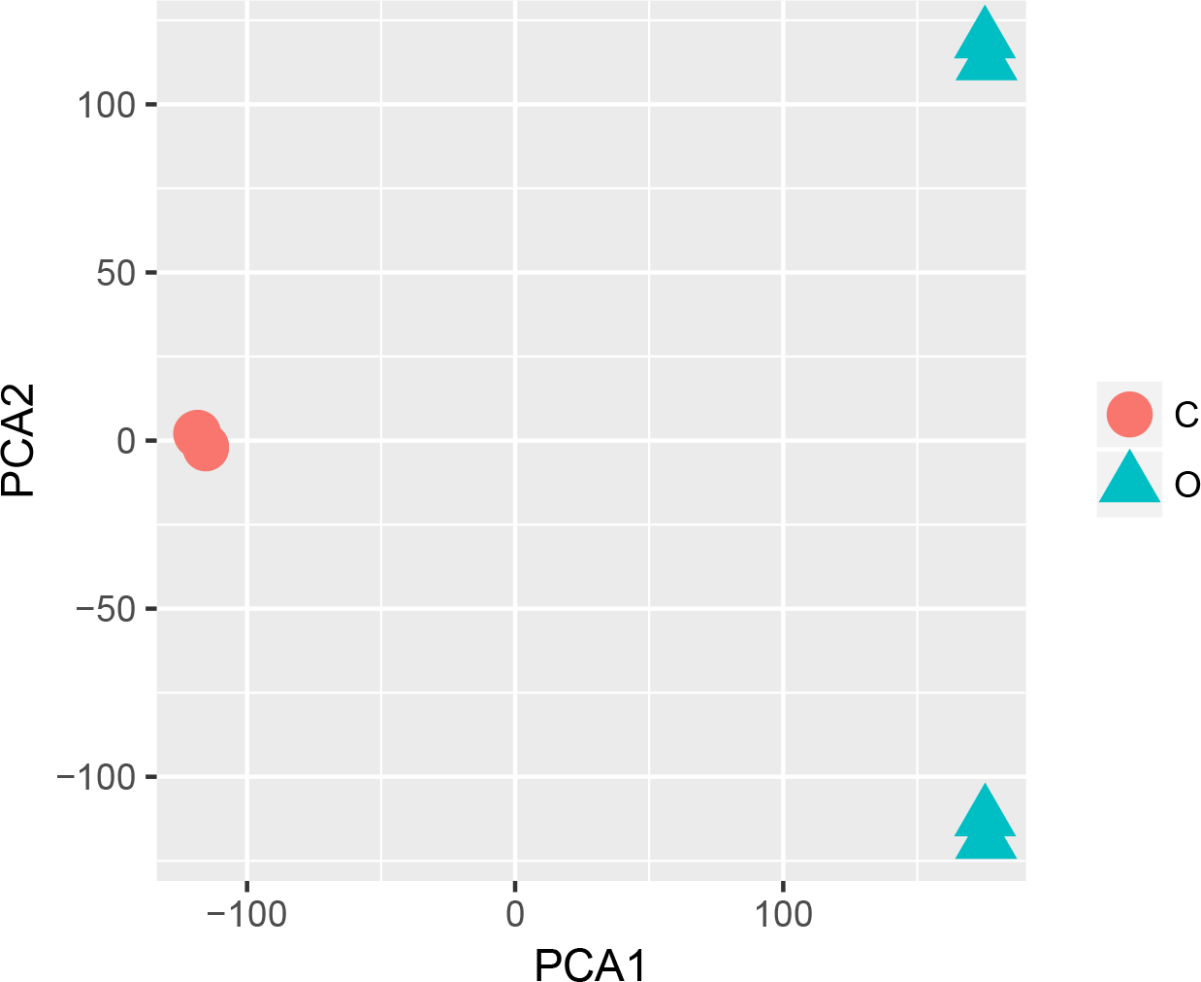
PCA plot.

**Figure 3:**
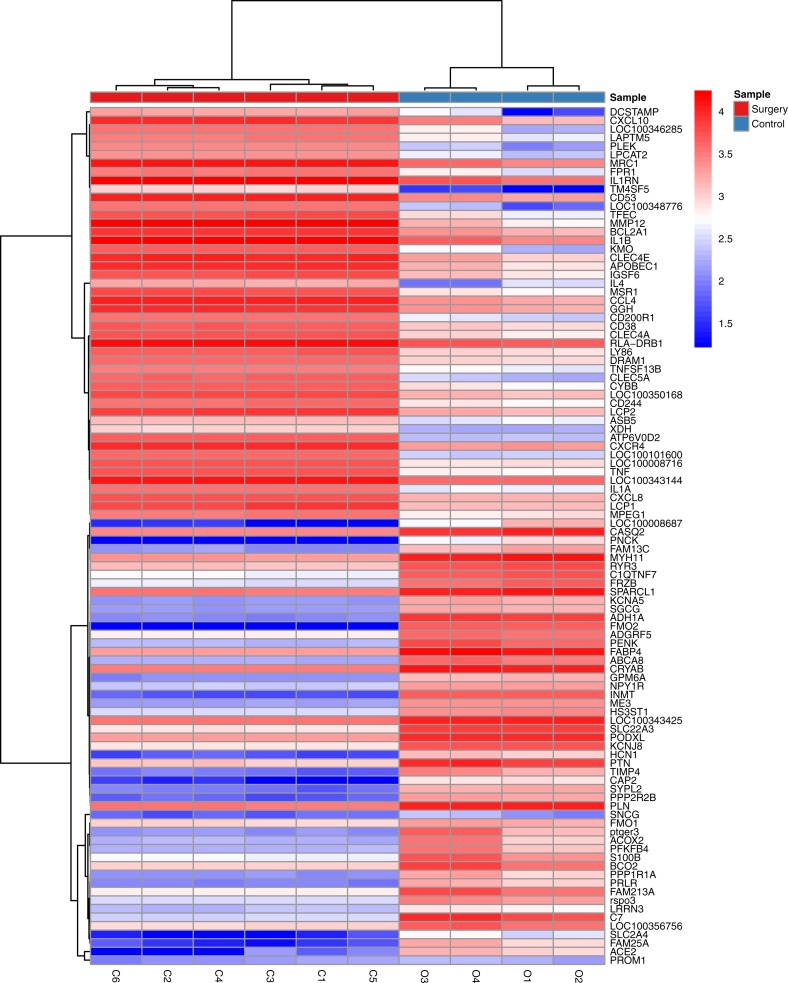
Hierarchical clustering analysis of top 100 DEGs.

The statistical analysis software R (version 3.4.1; R Core Team, 2017) was used to identify differentially expressed genes using an adjusted *p*-value <0.05 and —log2FC (fold change)—≥1 (Fig. 1) as criteria. A PCA plot was generated using normalized data. The PCA plot shows clear separation between the surgery and control group samples (Fig. 2). A total of 858 up-regulated and 817 down-regulated DEGs were obtained in the surgery vein graft group compared with the control group. Hierarchical clustering analysis of the top 100 DEGs is shown in Fig. 3.

### GO term enrichment analysis

The GO functional enrichment analysis showed that the up-regulated genes were significantly enriched in biological processes (BPs), including immune response, inflammatory response and innate immune response (Table 1). The down-regulated DEGs were significantly enriched in positive regulation of the ERK1 and ERK2 cascades, positive regulation of the apoptotic process, and chondrocyte differentiation (Table 2). For the cellular component (CC) category, the up-regulated DEGs were significantly enriched in extracellular exosome, extracellular space and membrane (Table 1). Interestingly, the down-regulated DEGs were also enriched in extracellular exosome, cytoplasm, and extracellular space (Table 2). For the molecular function (MF) category, the up-regulated DEGs were enriched in ATP binding, transmembrane signaling receptor activity, and kinase activity (Table 1), whereas the down-regulated DEGs were mainly enriched in calcium ion binding, heparin binding, and protein tyrosine phosphatase activity (Table 2).

**Table 1:** The top 5 enriched GO terms of the up-regulated DEGs.

Category	ID	Term	Gene count	*P* value
BP	GO:0006955	immune response	29	2.18E−08
	GO:0006954	inflammatory response	20	1.87E−04
	GO:0045087	innate immune response	19	6.05E−06
	GO:0043123	positive regulation of I-kappaB kinase/NF-kappaB signaling	14	0.014345
	GO:0010628	Positive regulation of gene expression	14	0.048003
CC	GO:0070062	extracellular exosome	183	3.16E−13
	GO:0005615	extracellular space	78	2.95E−10
	GO:0016020	membrane	54	0.017266
	GO:0005829	cytosol	48	0.037259
	GO:0005783	endoplasmic reticulum	34	0.002373
MF	GO:0005524	ATP binding	81	0.001476
	GO:0004197	cysteine-type endopeptidase activity	9	2.26E−04
	GO:0030246	carbohydrate binding	9	0.031484
	GO:0004888	transmembrane signaling receptor activity	7	0.006525
	GO:0016301	kinase activity	6	0.009682

**Notes.**

GO gene ontology BP biological process MF molecular function CC cellular component

*p*-value < 0.05 was considred as threshold values of significant differences.

**Table 2:** The top 5 enriched GO terms of the down-regulated DEGs.

Category	ID	Term	Gene count	*P* value
BP	GO:0070374	positive regulation of ERK1 and ERK2 cascade	12	0.024691
	GO:0043065	positive regulation of apoptotic process	12	0.027999
	GO:0002062	chondrocyte differentiation	8	3.70E−04
	GO:0006874	cellular calcium ion homeostasis	8	0.002072
	GO:0007224	smoothened signaling pathway	8	0.018141
CC	GO:0070062	extracellular exosome	123	0.002059
	GO:0005737	cytoplasm	118	0.013056
	GO:0005615	extracellular space	44	0.028228
	GO:0005925	focal adhesion	25	0.005367
	GO:0009986	cell surface	23	0.018489
MF	GO:0005509	calcium ion binding	38	0.001071
	GO:0008201	heparin binding	13	1.59E−04
	GO:0004725	protein tyrosine phosphatase activity	9	0.037067
	GO:0008144	drug binding	7	0.003298
	GO:0017046	peptide hormone binding	4	0.00765

**Notes.**

GO gene ontology BP biological process MF molecular function CC cellular component

*p*-value < 0.05 was considred as threshold values of significant differences.

### KEGG pathway analysis

In the KEGG pathway enrichment analysis, a total of 60 up-regulated pathways and 26 down-regulated pathways were obtained using the functional annotation tool DAVID. However, some enriched pathways evidently had no associations with VGR. For example, the most significant down-regulated pathway is pathways in cancer. Hence, a total of 14 potential key pathways were selected from these enriched pathways by literature review and general experience (Table 3); most of these pathways are involved in regulating cell proliferation, apoptosis, vascular smooth muscle contraction, and cell adhesion, which are processes that may be highly associated with VGR. Moreover, the 164 DEGs contained in these 14 key pathways were extracted and defined as the “KEGG genes” gene list.

**Table 3:** Potential key pathways selected out from KEGG pathway enrichment analysis of DEGs.

Category	Term	Count	*P* Value	Genes
Up-regulated	Cytokine-cytokine receptor interaction	32	4.04E−06	CSF2, IL1R2, TNFRSF21, TNF, CCL2, CCR1, CXCL8, IL15, IL7R, CCL4, IL17RA, CXCL10, TNFRSF11B, CXCR4, IFNG, IL1B, FAS, XCR1, LTB, IFNGR2, IL1A, IL4, IL18R1, IL6, TNFSF4, LTBR, TGFBR1, LOC100348776, CCR8, TNFSF10, TNFSF13B, CCR2
	TNF signaling pathway	19	1.11E−05	PIK3CG, CSF2, IL18R1, IL6, CCL2, TNF, MMP9, CREB1, IL15, MMP3, BIRC3, CXCL10, VCAM1, NOD2, MAPK14, IL1B, CREB3L1, FAS, TRAF3
	Cell cycle	19	3.93E−04	CDC6, CDK1, TTK, CHEK1, RB1, MCM4, YWHAE, MCM6, CCNE2, CCNE1, YWHAG, CCNB2, MCM7, MAD2L1, PCNA, BUB1B, ORC6, CCNA2, BUB3
	Toll-like receptor signaling pathway	15	5.71E−04	PIK3CG, IL6, TNF, LY96, TLR2, LOC100348776, CXCL8, TLR4, CCL4, CXCL10, IKBKE, CD80, MAPK14, IL1B, TRAF3
	DNA replication	8	0.002306	POLD3, MCM7, SSBP1, POLE, PCNA, MCM4, RPA3, MCM6
	Chemokine signaling pathway	19	0.007327	PIK3CG, LOC100008716, CCL2, VAV3, NCF1, HCK, CCR1, STAT5B, CXCL8, LOC100348776, CCL4, CXCL10, CCR8, CXCR4, CCR2, GNB4, XCR1, PLCB2, LOC100349255
	Cell adhesion molecules (CAMs)	16	0.017244	CADM1, SELL, NECTIN1, LOC100351865, VCAM1, ALCAM, CD80, RLA-DRB1, ICOS, ITGB7, CD274, LOC100350168, CD2, VCAN, LOC100343144, CD226
Down-regulated	cGMP-PKG signaling pathway	16	0.003361	ROCK1, GNAI1, MYLK3, MRVI1, ATP1A2, PRKG1, MYL9, AGTR1, EDNRB, KCNJ8, PLN, PDE5A, GUCY1A2, GUCY1B3, MYLK, PIK3R1
	TGF-beta signaling pathway	11	0.003933	BMP4, BMP2, LTBP1, ROCK1, ZFYVE9, LOC100008826, BMPR1B, MYC, BMP5, TGFB2, ACVR1C
	MAPK signaling pathway	20	0.004435	EGFR, FGFR1, MRAS, CACNB1, DUSP10, CACNB2, FGF10, CACNB3, FGF12, MECOM, TGFB2, MAP3K6, FOS, RASGRP3, DUSP1, MAP3K1, MYC, FGF2, GADD45A, CACNA1A
	Renin-angiotensin system	5	0.00852	AGTR1, AGTR2, ACE, ACE2, MME
	Vascular smooth muscle contraction	12	0.012552	AGTR1, ACTG2, ROCK1, MYLK3, CALD1, MRVI1, GUCY1A2, GUCY1B3, PRKG1, PLA2G2D, MYLK, MYL9
	Focal adhesion	16	0.01614	EGFR, CAV2, COL4A2, ROCK1, MYLK3, ITGA2, CAPN2, COL4A5, MYL9, VEGFC, ITGA6, ITGB8, PIK3R1, MYLK, THBS3, SHC4
	PI3K-Akt signaling pathway	22	0.016374	EGFR, FGFR1, COL4A2, RBL2, EFNA1, ITGA2, FGF10, FGF12, COL4A5, VEGFC, ITGA6, PRLR, ITGB8, EPOR, MTCP1, PPP2R2B, MYC, ANGPT2, FGF2, PIK3R1, THBS3, GHR

### PPI network analysis

**Figure 4:**
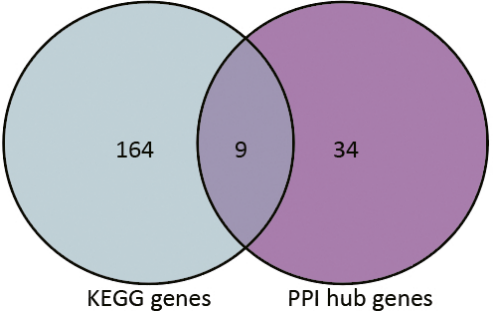
Venn diagram. 9 hub genes obtained from the intersection of gene list “KEGG gens” and “PPI hub genes”.

**Figure 5:**
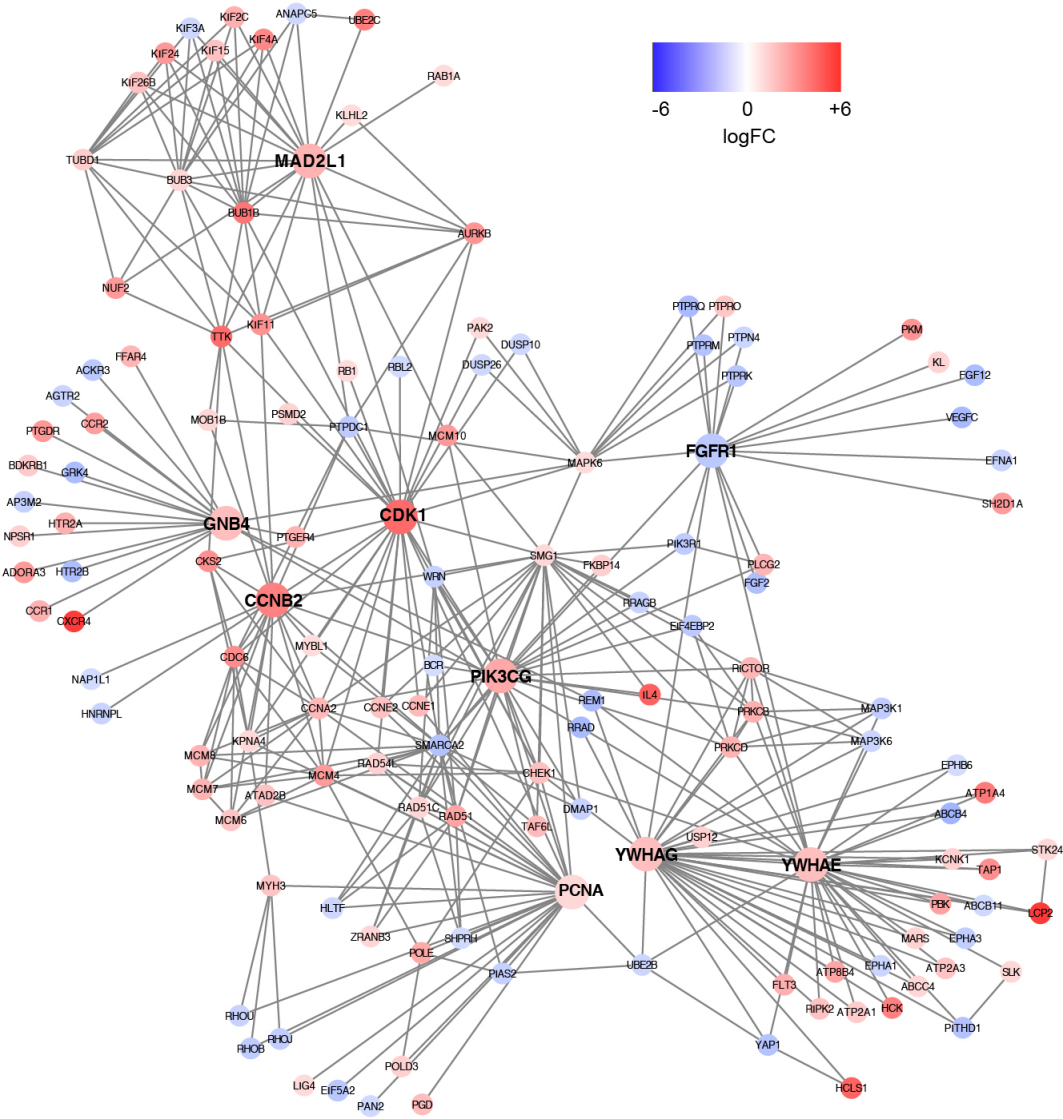
A comprehensive PPI network were constructed by merging nine PPI networks of the nine hub genes respectively. Each node corresponds to a DEG, and edges represent the interactions between DEGs. DEGs with higher degrees appear larger size. The gradual color from blue to red represents the changing process from down-regulation to up-regulation.

**Figure 6:**
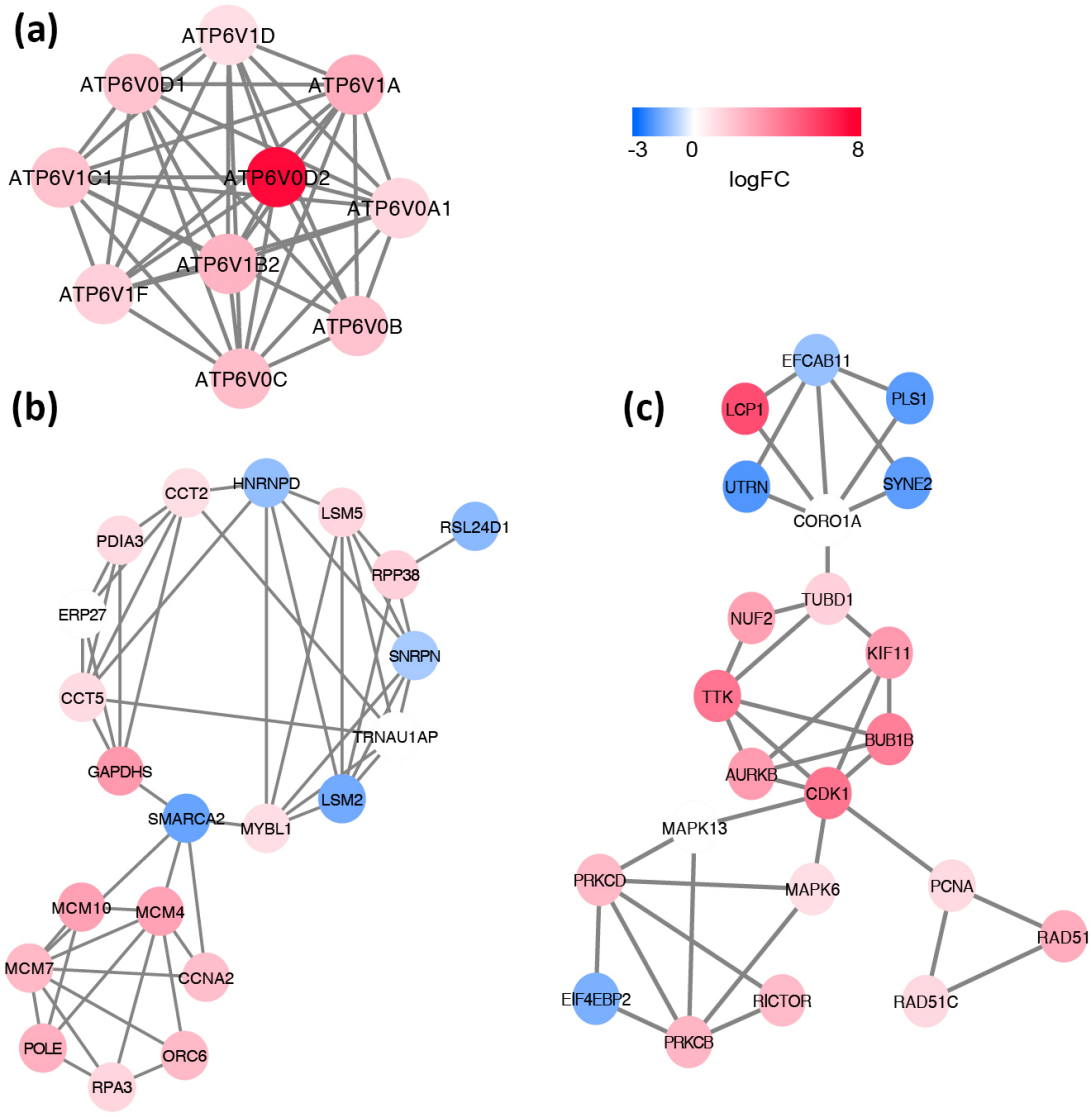
Significant modules identified from the whole PPI network by using MCODE app in Cytoscape. (A) module1, (B) module2, (C) module3. The gradual color from blue to red represents the changing process from down-regulation to up-regulation.

Based on the information in the STRING database, the PPI network consists of 695 nodes and 2,098 interactions. The top 34 hub nodes with more than 20 degrees were screened and defined as the “PPI hub genes” gene list. Moreover, nine hub genes obtained from the intersection of the “KEGG genes” and “PPI hub genes” gene lists (Fig. 4) are listed in Table 4. In addition, a comprehensive PPI network was constructed by merging the 9 PPI networks of the nine hub genes and visualized using the Cytoscape software (Fig. 5). A total of 695 nodes and 2,098 edges were analyzed using the plug-in MCODE, and the top three significant modules were screened (Fig. 6).

**Table 4:** 9 hub genes appearing higher degrees and involved in key pathways associated with VGR.

Gene	Degree	KEGG pathway	logFC	*P* Value
FGFR1	21	MAPK signaling pathway, PI3K-Akt signaling pathway	−1.59	5.13E−07
PIK3CG	24	TNF signaling pathway, Toll-like receptor signaling pathway, Chemokine signaling pathway	2.52	1.42E−13
CDK1	29	Cell cycle	4.34	9.07E−12
YWHAE	34	Cell cycle	1.36	2.57E−12
YWHAG	35	Cell cycle	1.89	1.41E−10
CCNB2	21	Cell cycle	3.70	2.87E−10
MAD2L1	21	Cell cycle	2.25	2.08E−13
PCNA	30	Cell cycle, DNA replication	1.16	2.51E−12
GNB4	20	Chemokine signaling pathway	1.91	5.52E−16

**Figure 7:**
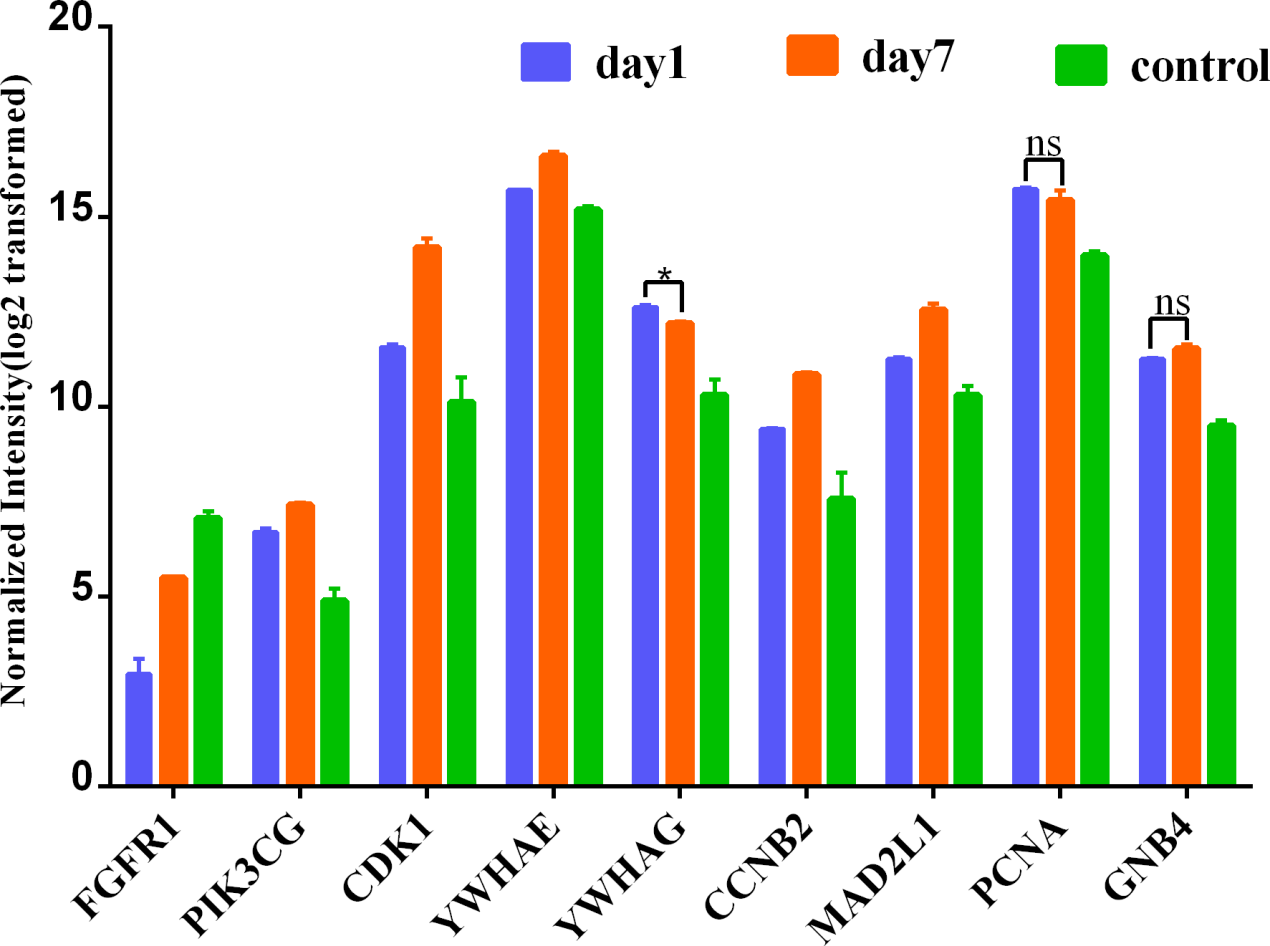
Expression of hub genes among 3 group. * denotes *P* > 0.05; ns denotes no statistical significance; all other comparisions : *P* < 0.001.

To further lend some more validity to the analysis results, the day 7 versus day 1 comparison was served as a control relative to day 7 versus sham surgery comparison. The expression of these nine hub genes in three groups (day 1, day 7 and control) is shown in Fig. 7. As expected, most hub DEGs identified in day 7 versus sham surgery group, are also differentially expressed in day 7 versus day 1 group, which may due to the influence of surgery hit, and all hub DEGs are also differentially expressed in day 1 versus sham surgery group.

## Discussion

Improving understanding of the cellular and molecular mechanisms underlying VGR is critically significant for reducing the rates of vein graft failure. In the present investigation, an integrated bioinformatics analysis was performed to identify potential key genes and pathways involved in regulating VGR. In summary, a total of 858 up-regulated and 817 down-regulated DEGs were screened. A total of 14 potential crucial pathways were selected from the functional annotation enrichment analysis. In the PPI network analysis, a comprehensive PPI network of the 9 hub genes was constructed. Furthermore, three significant modules were screened from the PPI network by module analysis.

**Figure 8:**
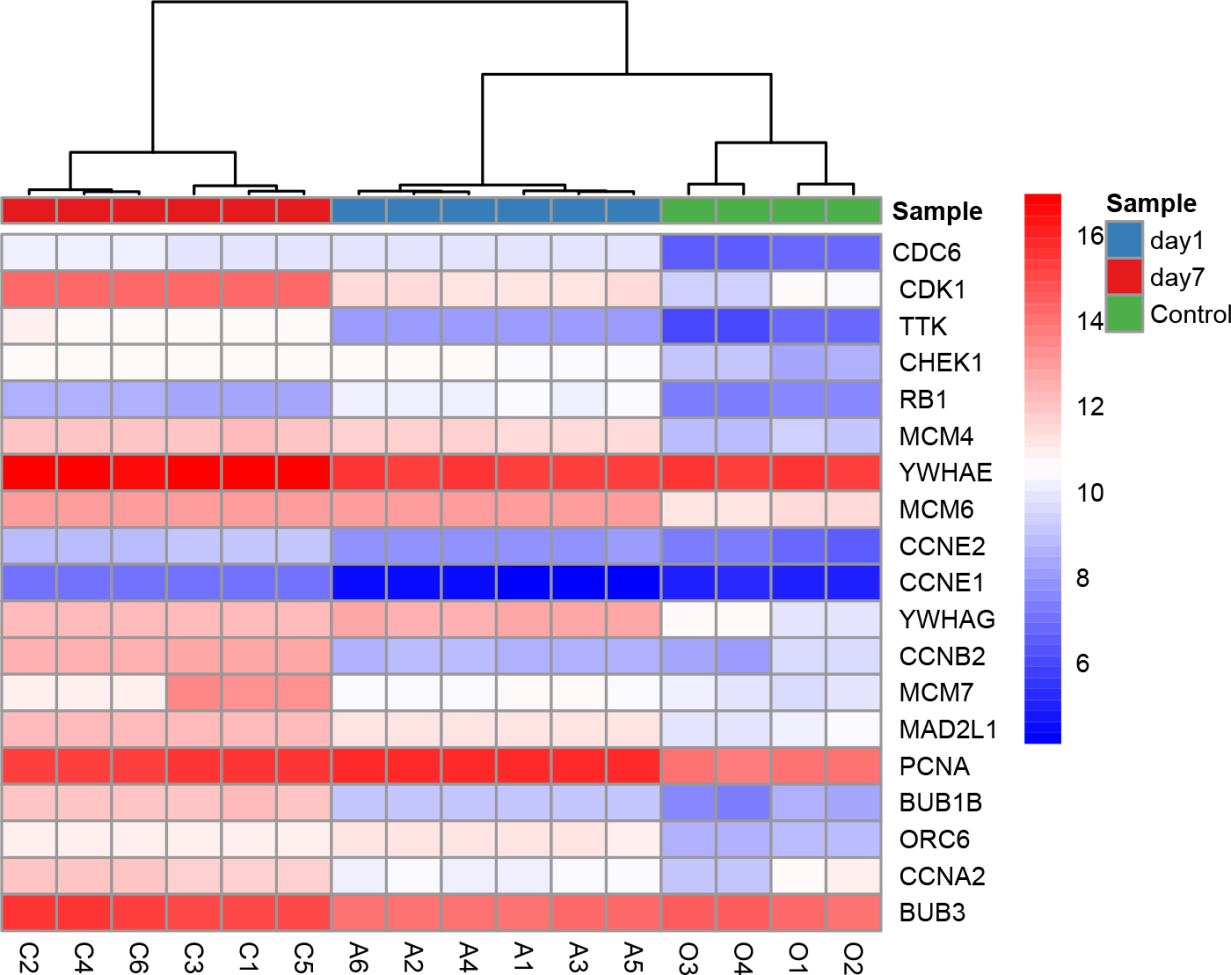
Hierarchical clustering analysis of DEGs involved in cell cycle pathway.

All nine hub genes showed higher degrees in the PPI network analysis and were involved in potential key pathways. Several previous studies demonstrated that fibroblast growth factor receptor 1 (FGFR1) activated the Akt/mTOR pathway (Chen & Friesel, 2009) and regulated phenotypic modulation of vascular smooth muscle cells (VSMCs) (Chen & Friesel, 2009; Chen, Simons & Friesel, 2009). As described in our previous study, the mTOR signaling pathway was indicated to play a major role in the arterialization of vein grafts (Wang et al., 2016). Moreover, a fibroblast growth factor receptor antagonist was validated to block growth factor-mediated VSMC proliferation by inhibiting binding of fibroblast growth factor 2 (FGF2) to VSMCs and soluble FGFR1 (Segev et al., 2002). VSMC proliferation was also thought to play a critical role in the pathophysiology of restenosis. The second hub gene PIK3CG (phosphatidylinositol-4,5-bisphosphate 3-kinase catalytic subunit gamma) was shown to be involved in three key pathways: the TNF signaling pathway, Toll-like receptor signaling pathway, and chemokine signaling pathway (Table 4). CDK1 (cyclin-dependent kinase 1) is a protein-coding gene that plays a key role in regulating the eukaryotic cell cycle by modulating the centrosome cycle and mitotic onset and thus cell proliferation. Cell proliferation requires the expression of CDKs, and CDKs are considered a proliferation signaling cascade of VSMCs (Schad et al., 2011). Evidence indicated that radiation inhibited VSMC proliferation via cell cycle arrest by enhancing p21 expression and suppressing CDK1 and 2 (Kim et al., 2004). Consistently, a derivative of the CDK inhibitor roscovitine was validated to potently induce G1 phase arrest and therefore inhibit VSMC proliferation (Sroka et al., 2010). YWHAE (tyrosine 3-monooxygenase/tryptophan 5-monooxygenase activation protein epsilon) has 34 degrees in the PPI network and is also included in the cell cycle pathway. Moreover, YWHAE belongs to the 14-3-3 family of proteins that induce signal transduction by binding to phosphoserine-containing proteins. Intriguingly, a recent study provided evidence that up-regulation of YWHAB in endothelial cells played a pivotal role in intimal hyperplasia following carotid artery injury via enhancing endothelial cell proliferation and migration (Feng et al., 2017). YWHAG knockdown in zebrafish was reported to reduce the brain size and increase the diameter of the heart tube (Komoike et al., 2010). CCNB2 (cyclin B2) is a member of the cyclin family, specifically the B-type cyclins. Evidence indicated that IL-18 promoted cell proliferation via NF-κB and the p38/ATF2 pathway by targeting CCNB2 (Zhang et al., 2015). Down-regulation of MAD2L1 was demonstrated to be involved in suppressing the proliferation, migration, invasion, apoptosis induction and cell cycle arrest of cancer cells (Li, Bai & Zhang, 2017). PCNA (proliferating cell nuclear antigen) is a molecular marker for proliferation due to its role in replication and is involved in both the cell cycle pathway and DNA replication; PCNA had 30 degrees in the PPI network. Previous studies showed that PCNA expression was correlated with stent-induced in-stent restenosis (Gao et al., 2013) and proposed that PCNA might be a potential future target (Wang, 2014). Guanine nucleotide-binding protein subunit beta-4 (GNB4) was involved in the chemokine signaling pathway and showed 20 degrees in the PPI network. Surprisingly, we noted that six of the nine hub genes (CDK1, YWHAE, YWHAG, CCNB2, MAD2L1, and PCNA) were involved in the cell cycle pathway, which further supported the enormous significance of this pathway in regulating VGR (Table 4). The key role of the cell cycle pathway in regulating VGR has been established (Sriram & Patterson, 2001); Hierarchical clustering analysis of DEGs in cell cycle pathways was shown in Fig. 8. However, the underlying molecular mechanisms remain largely unknown. Based on the abovementioned evidence, these hub genes may potentially interact with each other and regulate the VGR program by modulating the cell cycle pathway. Future studies focusing on revealing the specific cellular and molecular mechanisms involved in regulating the VGR program may provide novel therapeutic targets for VGR inhibition.

Over the past few decades, many powerful research tools in genomic technologies have advanced rapidly, such as genome sequencing and microarray technologies. Microarray technology has enhanced our ability to screen samples for thousands of genes at once, which may provide novel insights into potential molecular mechanisms involved in regulating the VGR program. Unfortunately, high throughput data associated with VGR are extremely scarce at present. “Restenosis” was used as a search term to search in the GEO (Gene Expression Omnibus) database (https://www.ncbi.nlm.nih.gov/geo/), and only 7 datasets were returned; this lack may be partly due to the challenge of obtaining appropriate samples *in vivo* and establishing a VGR animal model. Hence, we suggest that collaborations between clinicians and basic researchers may accelerate our understanding of the cellular and molecular mechanisms involved in VGR and therefore improve the clinical efficacy of occlusive artery disease.

Due to the extreme lack of high throughput data associated with VGR, more research is required to screen key genes and pathways involved in this process. Further experimental investigations are needed to estimate the role of these candidate genes and pathways. However, there are certain limitations in the present study, the aforementioned results, including the gene expression levels and their functions, were needed to be validated by experiments, which would be carried out in our further studies.

## Conclusions

In conclusion, this study provides a set of potential therapeutic targets for future investigations into the molecular mechanisms involved in VGR. However, we acknowledge that there were certain limitations in this study. The aforementioned results, including the expression level of genes and the definite functions of potential hub genes and key pathways, were needed to be validated by experiments, which would be conducted in our further studies.

##  Supplemental Information

10.7717/peerj.4704/supp-1 Supplemental Information 1 Raw data and code Raw data and R code used in this manuscript.

10.7717/peerj.4704/supp-2 Supplemental Information 2 86 KEGG pathways

10.7717/peerj.4704/supp-3 Supplemental Information 3 Single DEGs KEGG pathways

## Additional Information and Declarations

### Data Availability

The following information was supplied regarding data availability: The raw data and code are provided in a Supplemental File.
